# Complete Genome Sequence of SfII, a Serotype-Converting Bacteriophage of the Highly Prevalent *Shigella flexneri* Serotype 2a

**DOI:** 10.1128/genomeA.00626-13

**Published:** 2013-09-12

**Authors:** Divya T. George, David P. Stephenson, Elizabeth Tran, Renato Morona, Naresh K. Verma

**Affiliations:** Division of Biomedical Science and Biochemistry, Research School of Biology, the Australian National University, Canberra, Australian Capital Territory, Australiaa; School of Molecular and Biomedical Science, the University of Adelaide, Adelaide, South Australia, Australiab

## Abstract

SfII is a serotype-converting temperate bacteriophage of the highly prevalent *Shigella flexneri* serotype 2a. We isolated the SfII phage from a wild-type strain of *S. flexneri* serotype 2a. Here, we present the complete genome sequence of this phage.

## GENOME ANNOUNCEMENT

*Shigella flexneri* strains are most frequently linked with outbreaks of shigellosis, and these strains are susceptible to infection by temperate lambdoid bacteriophages. Here, we isolated and completely sequenced bacteriophage SfII, induced from a virulent *S. flexneri* 2a serotype, strain NCTC 4. *S. flexneri* 2a is the most widespread serotype associated with shigellosis cases (1).

Electron microscopy data (2) show that SfII morphologically resembles bacteriophages belonging to group A1, from the family *Myoviridae* and the order *Caudovirales* (3). SfII confers its host with the serotype-converting O-antigen-modifying glucosyltransferase (*gtr*) genes. Serotype conversion is a key defense mechanism used by *S. flexneri* to evade host defense responses (4). Not much is known about temperate bacteriophages of *S. flexneri* outside their role in serotype conversion*.*

To further our understanding of *S. flexneri* phages, the complete sequence of SfII was determined. Eight PstI fragments generated from the SfII genome were cloned individually into a pBlueScript vector, and both strands of each fragment were then sequenced using Sanger sequencing. Primer walking was used to fill in gaps and determine the order of the fragments. The DNA sequences were assembled into contigs using BioEdit (5). Putative open reading frames (ORFs) were identified using the NCBI ORF Finder and CLC Main Workbench version 6.5.

The genome of SfII is linear, double-stranded, and is 41,475 bp with an average G+C content of 49.17%, corresponding to 58 coding sequences (CDSs). Most of the genome (71.57%) is predicted to be transcribed from the sense strand, while 19.14% of the genome, including the *gtr* cluster, is predicted to be transcribed by the antisense strand. In database searches of the 58 predicted ORFs using NCBI BLASTx, 41 were assigned functions and 17 were identified as hypothetical proteins.

Nucleotide and protein homology searches with other published *S. flexneri* phage genomes indicate that SfII, like SfV (6), SfI (7), and Sf6 (8), is a member of the temperate lambdoid group of bacteriophages with their conserved arrangements of early and late genes. A comparison of phage terminases found that the packaging mechanism used by SfII is similar to those of SfV and SfI, with cohesive end (*cos*) sites spanning bp 59 to 125, adjacent to the terminases. Although SfII contains features of SfV and SfI, it has a unique host range. While SfV was capable of infecting 7 of the 12 *S. flexneri* serotypes tested (serotypes 1a, 1b, 2a, 2b, 3b, 4b, and Y), SfII and SfI only infected 3 (serotypes 3b, 5a, and Y) and 2 (serotypes X and Y) of the 12 serotypes tested, respectively.

The main differences between *S. flexneri* SfII, SfV, and SfI lie in their immunity, replication, and Nin regions. Our analysis found the following genes to be unique to SfII: SfII_25 (acyltransferase), SfII_27 (*gtrII*), SfII_26, SfII_55, and SfII_56 (transposases), SfII_35, SfII_36, SfII_40, SfII_41, SfII_49, SfII_ 50, and SfII_57 (hypothetical proteins), and SfII_48 (antiterminator Q). It will be interesting to study the hypothetical proteins that are unique to SfII in the context of *Shigella* pathogenesis.

### Nucleotide sequence accession number.

The complete genome sequence of bacteriophage SfII has been deposited in GenBank under the accession no. KC736978.
